# Editorial: Computational Tools in Inferring Cancer Tissue-of-Origin and Molecular Classification Towards Personalized Cancer Therapy, Volume II

**DOI:** 10.3389/fgene.2021.735103

**Published:** 2021-08-04

**Authors:** Ling Kui, Wenzhe Mao, Aasim Majeed, Jian Chen

**Affiliations:** ^1^Shenzhen Qianhai Shekou Free Trade Zone Hospital, Shenzhen, China; ^2^Harvard Medical School, Dana-Farber Cancer Institute, Brookline, MA, United States; ^3^School of Pharmacy, Jiangsu University, Zhenjiang, China; ^4^Molecular Genetics Laboratory, Department of Botany, Central University of Punjab, Bathinda, India; ^5^International Genome Center, Jiangsu University, Zhenjiang, China

**Keywords:** cancer, molecular classification, computational tools, machine learning, cancer therapeutics

With the advancement of sequencing technologies, there has been a rapid accumulation of data making it difficult to decipher the key genetic elements, their nature, and alterations for progression or regression of cancers. Further, enlightening an optimum gene set for optimal classification, diagnosis, and prognosis of cancer types is difficult without reliable and efficient computational tools. Progress in bioinformatics tools is parallel with sequencing data accumulation. Computational tools are being continuously refined and developed to meet specific challenges in cancer biology, the primary problem with them is that their accuracy is often insufficient for clinical use (Kui et al., 2021). As the molecular classification, appropriate diagnosis and prognosis, and markers for cancer improve better, more strategic novel drugs and efficient cancer treatments can be developed. Computation tools would play a great role in this direction.

In this editorial, we presented an account of how computational tools have greatly facilitated in unearthing the classification, prognosis, and therapeutic treatments of different cancer types. This editorial is based on 12 research articles, which sheds light on the power of computational tools to reveal the novel targets for cancer therapy and enhance the survival of patients with but not limited to glioblastoma, ciliated muconodular papillary tumors, adenosquamous carcinoma (ASC), breast cancer, esophageal cancer (EC), in colorectal cancer (CRC), metastatic melanoma, and multiple myeloma. The computational pipelines developed in these studies have a great potential to be extended to uncover novel therapeutic targets of other cancer types.

Four studies focused on the usage of specific computational approaches to address diverse problems. Chen et al. proposed to use machine learning algorithms to identify primary lesions for primary metastatic tumors. The fundamental idea behind their model is that different tumor types exhibit specific expression profiles for certain genes, which could be captured through machine learning models to classify the primary lesions. In essence, they used gene expression data from TCGA and GEO, analyzed and processed it to obtain a relatively suitable machine learning model followed by evaluation of the efficiency of diagnosis of primary lesions. They used XGBoost for classification and their results revealed that by combining tumor data with machine learning methods, the classification of different cancers can be achieved with specific accuracy, which can be used to predict the location of primary metastatic tumors. Cui et al. developed a novel pipeline, which not only compares two single-cell clusters but also calls for differential gene expression, coexpression network modules, etc. They used two single-cell data sets; Usoskin from the GEO database and Xin dataset of the human pancreas. Different types of analysis were then performed sequentially through a variety of computational tools to create a smooth pipeline. The pipeline implements DEsingle and SigEMD for differential gene expression analysis, DGCA for differential correlation analysis, WGCNA for network analysis, and DNA for differential network analysis. This pipeline is very effective to unravel the key differences between cell clusters and cell types and provides one place for easy computational analysis of single-cell data sets. Zhao et al. designed an Autoencoder-based computational framework, which could capture both intrinsic and extrinsic features of melanoma. They used the expression data of the TCGA metastatic melanoma gene RNA-seq dataset from Firehose and decomposed it into a small number of representative nodes. Further, microarray datasets from GEO for melanoma were used for prognosis analysis. They identified many nodes that were significantly associated with the prognosis of melanoma patients using Cox proportional hazard models. A tumor-intrinsic (TI) signature and a tumor-extrinsic (TE) signature were established from the two most prognostic nodes. Both these signatures highly predicted the patient's overall survival. In addition, the TE signature successfully predicted the response of patients to immunotherapy techniques. Using an integrative approach of somatic mutations and gene expression data, Jiang and Jin proposed a novel method for the identification of breast cancer-associated mutated genes. The fundamental theme behind their analysis is to first create a mutation matrix data and evaluating mutation frequency for each gene, then to create a gene expression matrix with expression values for each gene. Finally, both data sets are mapped to identify the co-expression profile. Their results indicated that this integrative approach is effective in breast cancer classification.

Two studies focused on the classification and prognosis of glioblastoma multiform (GBM), which lacks accurate prognostic markers and drug targets. Yuan et al. aimed to create a new molecular classification and to provide new therapeutic targets for GBM. They performed an integrated analysis based on the SNPs, DNA copy, DNA methylation, and mRNA expression profile data of 117 patients. The data was obtained from the TCGA database and Genomic Data Commons database (GDC). MutSigCV and GISTIC modules from GenePattern were used in the analysis of driver genes and landmark CNV events in GBM, respectively. Using the cluster of cluster analysis (CoCA), they found two novel subtypes, HX-1 and HX-2 depicting three variable methylation positions and fifteen gene mutations. These subtypes may act as potential prognostic biomarkers for patients with glioblastoma. Zhang et al. tried to classify glioblastoma subtypes on the basis of different degrees of gene methylation. They used the methylation datasets from Gene Expression Omnibus (GEO), identified the methylation loci, which served as potential biomarkers to classify and annotate the different GBM subgroups. They used powerful machine learning algorithms to achieve their goals. Monte Carlo feature selection (MCFS) and incremental feature selection (IFS) methods were used to extract 4,100 essential methylation sites and support vector machine (SVM), random forest (RF) metaclassifier, and repeated incremental pruning to produce error reduction (RIPPER) were used during classification. Functional enrichment analysis of these dysmethylated genes using GO and KEGG databases revealed several biological functions related to GBM classification.

Three studies aimed to reveal the novel prognosis-related signatures in different cancers. Esophageal cancer (EC) is a global fatal disease with a poor prognosis. Huang et al. aimed to evaluate the significance of genetic alteration (*CDK4* amplification) in the prognosis of esophageal squamous cell cancer (ESCC). Through tissue microarray and fluorescence *in situ* hybridization they found that among the investigated 520 patients with ESCC, 8.5% exhibiting *CDK4* amplification showed a negative correlation with disease progression and significantly better survival. Thus, they declared *CDK4* amplification as an independent prognostic factor for the survival of patients with ESCC. With the aim of identifying the novel DNA damage and repair-related prognostic genes in colorectal cancer (CRC), Wang et al. identified 1,545 genes related to DNA damage and repair. They used gene expression data of 471 COAD (Colon adenocarcinoma) and 41 normal samples from The Cancer Genome Atlas-Colon adenocarcinoma (TCGA-COAD) and 4 datasets of colon cancer from the GEO database. Following the gene set enrichment analysis (GSEA), the prognostic relevance of the individual genes was evaluated through Cox regression analysis on the TCGA-COAD dataset. A set of 12 genes related to DNA damage-and-repair were identified, which classified COAD patients into high and low-risk groups. Genes co-expressing with these 12 genes were identified through Pearson's correlation method. WGCNA with Topological Overlap Matrix (TOM) was used to construct the gene-coexpression network. Functional annotation of the functional gene modules was carried out using Gene Ontology (GO) and Kyoto Encyclopedia of Genes and Genomes (KEGG). The gene set identified in this study has great potential in the prognosis, and treatment of CRC. Further, using The Cancer Genome Atlas (TCGA)-Multiple Myeloma Research Foundation (MMRF) dataset as a training dataset, Wang et al. analyzed the expression profiles through R package limma and evaluated the prognostic relevance of each gene through univariate Cox regression. Risk predictions were established through Lasso and stepwise Cox regressions followed by validation using GEO datasets. A set of eight RBP hub genes were identified, which classified multiple myeloma patients into high- and low-score groups. Functional analysis through Gene Ontology, KEGG Enrichment Analysis, and Gene Set Enrichment Analysis (GSEA) revealed that the major pathway through which RBP's could lead the development of myeloma may be the spliceosome pathway.

Two studies identified mutations in driving cancers. Yang et al. used the whole exon sequencing and immune checkpoint analysis of five patients with ciliated muconodular papillary tumors (CMPT) to elucidate the molecular details and histogenesis in CMPT. They observed 77 gene mutations in the patient's tumor tissue and 31 mutations in the border tissue. Interestingly, CMPT shared the same phylogeny with cancer tissue. These results suggest the CMPT indeed are neoplastic processes with immune escape and have malignant potential. Recent studies have revealed that the clinical outcome of multiple cancers could be predicted through tumor mutational burden (TMB). To ascertain the relationship between TMB level and clinical features and outcomes of lung Adenosquamous carcinoma (ASC), Cheng et al. used NGS and immunohistochemistry approach and identified 95 unique genes with somatic variations from a total of 475 genes evaluated. TMB was found to be associated with pathological stages, invasion of lymph node, and overall survival but not with age, sex, smoking history, and tumor size in lung ASC. Moreover, no correlation between TMB and mutations in *TP53* and *EGFR* was observed. This study, therefore, provided an evidence that higher TMP correspond to lesser survival and higher lymph node invasion.

One study focused its interest on improving the existing medical imaging technology, which is a commonly useful approach in disease diagnosis and progression. With rapid advancements in deep learning, medical imaging technology has been revolutionized. Most medical imaging techniques involve encoder-decoder system, the classical architecture of which is implemented in U-Net. Several modified versions of U-Net have been introduced till now, all of which have two major limitations; loss of diversity features caused by fixed receptive field of the convolution kernel and loss of information when a single convolutional sequence is used in extracting features at each scale. With the aim of overcoming these limitations, Su et al. developed a new version of U-Net called multiscale U-Net (MSU-Net), which employed a new image segmentation architecture. It is based on Multi-scale blocks composed of convolution sequences with different receptive fields, which facilitates extraction of more information with diversified features. Their results showed that MSU-Net enabled significant improvement of semantic segmentation. MSU-Net integrates multiple convolution sequences having receptive fields of different sizes, which produces more conspicuous object features during forward propagation. Besides, MSU-Net is flexible enough to be integrated with other network structures. MSU-Net showed improved results, with 5-fold cross validation when applied on five biomedical image segmentation datasets; (1) 30 serial section Transmission Electron Microscopy (ssTEM) images (512 × 512 pixels) of the first instar larva ventral nerve cord (VNC) of the *Drosophila*, (2) The Breast Ultrasound Dataset B (BUL) comprising of 163 ultrasound images (760 × 570 pixels) of breast lesions, (3) 800 Chest X-ray (CXR) images (4,456 × 4,456 pixels) from the standard digital image database for Tuberculosis, (4) 2,594 RGB images of skin lesions (2,166 × 3,188 pixels), and (5) Nuclei Segmentation (NS) dataset from The Cancer Genome Atlas (TCGA) comprising of 30 digitized Hematoxylin and Eosin-stained frozen sections (512 × 512 pixels). This imaging technology may perform better in tracing, diagnosis, prognosis, and possible treatment of different cancer types.

## Author Contributions

This editorial was designed by LK and written by LK and AM. WM and JC revised the editorial. All authors made a direct and intellectual contribution to this topic and approved the article for publication.

## Conflict of Interest

The authors declare that the research was conducted in the absence of any commercial or financial relationships that could be construed as a potential conflict of interest.

## Publisher's Note

All claims expressed in this article are solely those of the authors and do not necessarily represent those of their affiliated organizations, or those of the publisher, the editors and the reviewers. Any product that may be evaluated in this article, or claim that may be made by its manufacturer, is not guaranteed or endorsed by the publisher.
